# Outcomes of prostate cancer patients after robot-assisted radical prostatectomy compared with open radical prostatectomy in Korea

**DOI:** 10.1038/s41598-023-34864-8

**Published:** 2023-05-15

**Authors:** Jaehun Jung, Gi Hwan Bae, Jae Heon Kim, Jaehong Kim

**Affiliations:** 1 Department of Preventive Medicine, Gachon University College of Medicine, Incheon, South Korea; 2 Artificial Intelligence and Big-Data Convergence Center, Gil Medical Center, Gachon University College of Medicine, Incheon, South Korea; 3 Department of Urology, Soonchunhyang University Hospital, Soonchunhyang University Medical College, Seoul, South Korea; 4 Urological Biomedicine Research Institute, Soonchunhyang University Seoul Hospital, Seoul, South Korea; 5 Department of Biochemistry, College of Medicine, Gachon University, Incheon, 21999 Republic of Korea

**Keywords:** Medical research, Cancer

## Abstract

Limited evidence exists regarding the value of robot-assisted radical prostatectomy (RARP) in promoting health outcomes in patients with prostate cancer (PCa) in Korea, prompting a study to determine its clinical impact. The study included 15,501 patients with PCa who underwent RARP (n = 12,268) or radical prostatectomy (RP) (n = 3,233) between 2009 and 2017. The outcomes were compared using a Cox proportional hazards model after propensity score matching. Hazard ratios of all-cause overall mortality after RARP compared to that after RP within 3 and 12 months were (6.72, 2.00–22.63, *p* = 0.002) and (5.55, 3.31–9.31, *p* < 0.0001), respectively. The RARP group in four hospitals with the largest PCa surgery volume during the study period had worse percentile deaths than the total RARP patients within 3- (1.6% vs. 0.63%) and 12-month post-op (6.76% vs. 2.92%). The RARP group showed specific surgical complications, like pneumonia and renal failure, more than the RP group. A significantly higher short-term mortality and only modestly lower surgical complications occurred in RARP than RP group. RARP performance status may not be superior to that of RP as previously reported and perceived, possibly due to increased robotic surgery in the elderly. More meticulous measures are needed for robotic surgery in the elderly.

## Introduction

Despite the growing concerns and various recent warnings that the actual benefit of robot-assisted radical prostatectomy (RARP) use is unclear^1,2^, it is frequently used for localized prostate cancer (PCa)^3^. In the United States, it is the most common surgical approach for PCa^4^, and by 2014, it accounted for up to 90% of the total radical prostatectomies (RP) conducted^5^.

Currently, in Korea, more than 3,300 cases of urologic robotic surgeries are performed annually^6^, with PCa as the top indication for the da Vinci surgical system (Intuitive Surgical, Mountain View, CA, USA) use. In Korea, RARP was introduced in 2005^6^ and by 2013, its use had exceeded that of the conventional RP^7^. Other than for robotic colorectal surgeries^8^, evidence on the value of RARP practice based on any large-scale real-world clinical cohort study involving several participating hospitals is lacking. Indeed, most studies indicate that the benefits of RARP practice are limited to short-term outcomes such as less blood transfusions, less days of hospitalization, and wound complications, other than improved survival rates, in comparison with a conventional alternative like RP.

This study aimed to determine the performance status of RARP in comparison with that of RP in Korea using the National Health Insurance Services (NHIS) patient reimbursement data.

## Materials and methods

### Data source

The NHIS is a universal single payer obligatory insurance plan that covers approximately 98% of all Korean nationals and long-term residents; it reimburses all the covered medical costs^9,10^. The NHIS data included in this study were age, sex, healthcare use (clinic, hospital, and emergency department visits), diagnoses coded according to the International Classification of Diseases and Related Health Problems, 10th edition (ICD-10), and prescription of medications and procedures covered by the NHIS.

### Study population

Customized data of PCa patients who underwent RP, or RARP procedures from January 1, 2009, to December 31, 2017, were extracted, and the patients were followed-up until December 31, 2017. The incidence of RARPs was determined and validated by a comparison with the National Evidence-based Healthcare Collaborating Agency (2014) and Intuitive Surgical Korea’s reported incidence of RARPs^6^. RP was identified using Korean electronic data interchange (EDI) procedure codes for prostatectomy (code “R3960,” “R3950”). We defined RARP, operationally, as the absence of a surgery code despite the presence of a general anesthesia code and a postoperative pathology examination code. Patients with a diagnosis of PCa before 2009, and patients with other cancer diagnosis before PCa were excluded. Lastly, we excluded those patients who underwent RP, or RARP before 2009 or patient who experienced surgical events before the diagnosis of PCa.

### Primary and secondary outcomes

The study primary outcome was short-term all-cause overall mortality (OM) observed during the follow-up period (between operation date and December 31, 2017) while major complication after surgery was the secondary outcome (Table S1). The outcome was grouped based on the time of occurrence into within 3 and 12 months after surgery. The ICD-10 code corresponding to the occurrence of each surgical event was assigned after surgery. We also divided our observation periods into early (2009–2012) and late (2013–2017).

### Statistical analysis

Baseline characteristics were compared according to the surgery type. Continuous variables are expressed as means ± standard deviation. As multiple testing was performed between subgroups, we set a robust cutoff of *P* < 0.01 for statistical significance. To address possible selection bias owing to differences in the proportion of basic characteristics between surgery types, propensity score matching (PSM) was used. Propensity scores were defined as the probability of a patient undergoing RP and RARP, and matching was performed. The score was calculated using multiple logistic regression based on age, chemotherapy, hormone-therapy, radiation therapy (Table S2), year of operation, socioeconomic level, Charlson comorbidity index (CCI) (Table S3), and relative level of operating hospital. We used a greedy nearest neighbor matching on the logit of the propensity score. CCI scores were calculated using the 1-year data before PCa diagnosis.

After PSM, Cox proportional hazards models were used to determine association between surgery types and all-cause mortality, and hazard ratios (HR) and 95% confidence intervals (CI) of the association between types of surgeries and complications were reported. SAS, version 9.4 (SAS Institute Inc, Cary, NC), and R, version 3.5.2 (R Foundation for Statistical Computing, Vienna, Austria), were used for the analyses. J.J. and G.W.B. had full access to all study data and were responsible for data integrity and data analysis accuracy.

### Ethical approval

All experimental protocols were approved by the Institutional Review Board of Gachon University Gil Medical Center (IRB No. GCIRB2018-380), and participants Informed consent was waived by the ethics committee of Gachon University Gil Medical Center because the data involved routinely collected medical data that was processed anonymously at all stages. All study methods were carried out based on the Declaration of Helsinki.

## Results

### Participant characteristics

**Figure 1:**
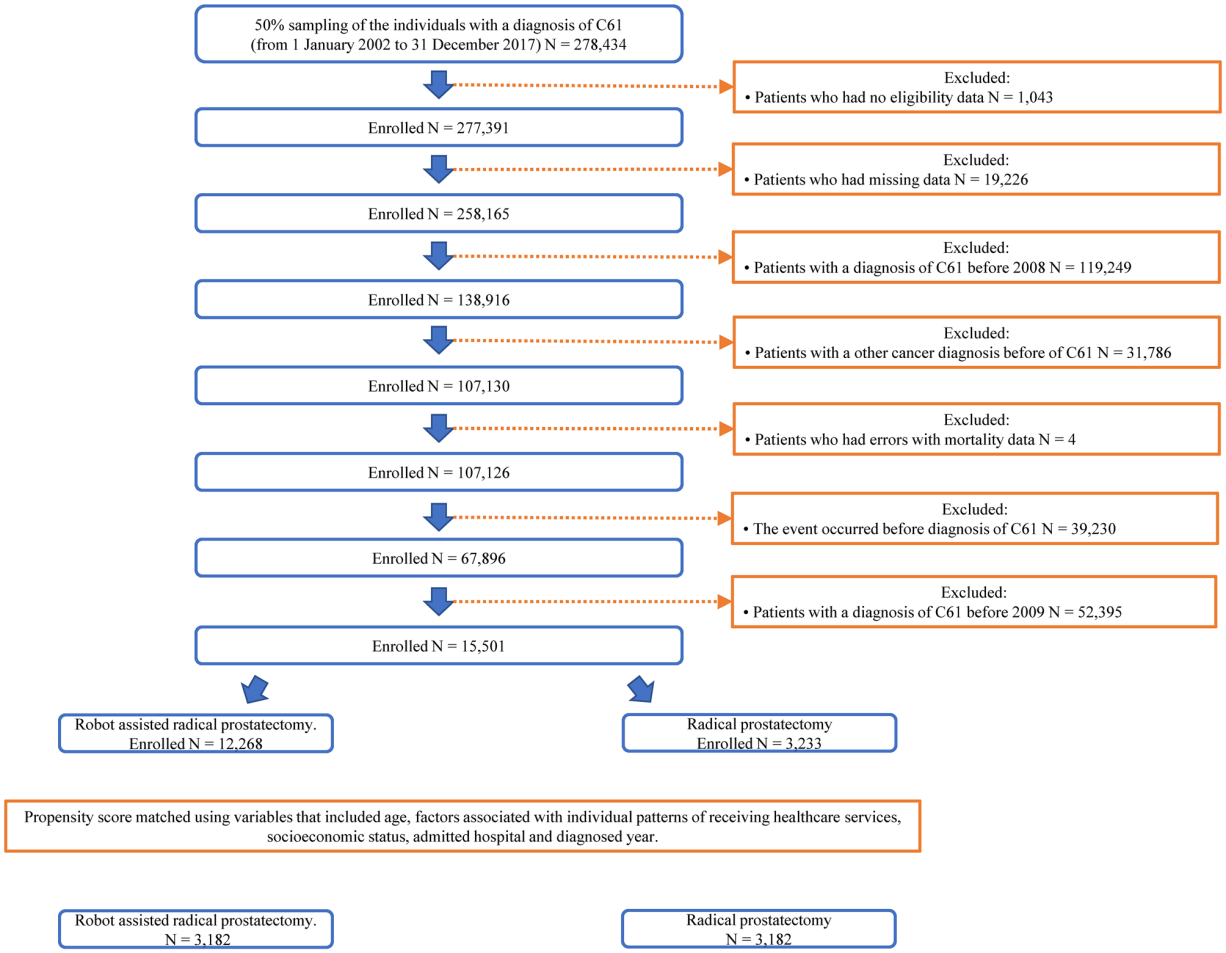
Flowchart of participant selection.

The exclusion criteria for selecting PCa patients are shown in Fig. 1. This study included 3,233, and 12,268 patients who underwent RP and RARP between 2009 and 2017, respectively. The summary of patients before and after PSM is shown in Table 1.

**Table 1:** Study subject analysis before/ after PSM.

Variable		Before PSM			After PSM			
		RARP	RP	*p*	RARP	RP	*p*	SMD
	Subject number	12,268	3233		3182	3182		
Age	Mean age	67.98 ± 9.36	66.83 ± 6.35	< .0001	66.89 ± 6.22	66.89 ± 6.22	1.0000	0
	30 ~ 39 YO	32	0	< .0001	0	0	1.0000	
	40 ~ 49 YO	235	18		13	13		
	50 ~ 59 YO	2017	378		369	369		
	60 ~ 69 YO	4487	1649		1623	1623		
	70 ~ 79 YO	4227	1165		1157	1157		
	80 ~ YO	1265	22		20	20		
Operation year	2009	1039	391	< 0.0001	340	385	0.1508	
	2010	1105	369		366	368		
	2011	1142	465		394	443		
	2012	1134	424		417	415		
	2013	1261	403		400	399		
	2014	1382	351		352	351		
	2015	1439	316		326	316		
	2016	1744	266		306	261		
	2017	2022	248		281	244		
Insurance premium level	1	803	268	< .0001	237	260	0.4646	
	2	684	244		251	234		
	3	668	245		211	234		
	4	651	187		181	187		
	5	778	218		217	214		
	6	888	257		236	251		
	7	1109	328		316	326		
	8	1483	409		450	408		
	9	2010	495		461	494		
	10	3194	582		622	574		
CCI		1.45 ± 1.52	1.45 ± 1.49	0.4471	1.47 ± 1.52	1.46 ± 1.48	0.6825	0.01
Distribution of surgical events (I)	in top tier academic centers*	11,587	3232	< .0001	3182	3182	1.0000	
	Others	681	1		0	0		
Distribution of surgical events (II)	Big 4 hospitals**	3478	726	< .0001	644	716	0.0277	
	Others	8790	2507		2538	2466		

PSM, propensity score matching; RARP, robot-assisted radical prostatectomy; RP, radical prostatectomy; YO: years old.

* In Korea, the actual number of top tier academic medical centers varies slightly depending on how many get designated as “general hospital for the treatment of severely ill patients” every 3 years. Thereby, we presented their approximate number as 40.

** Big 4 hospitals: Four academic medical institutes that had the largest volume of PCa surgeries during our entire observation period.

In Korea, every 3 years, approximately 40 general hospitals affiliated with medical schools and designated as “general hospital for the treatment of severely ill patients” are rated as top-tier academic medical centers. Most of the RP, and RARP cases occurred in these top-tier academic medical centers (Group I in Table S4). 22.45%, and 28.35% of total RP, and RARP cases, respectively, were performed in four top-tier academic medical centers with the largest volume of PCa surgeries during the study observation period (Group II in Table S4). We call these four hospitals as ‘Big 4’.

### Comparison of the primary outcomes between groups

The RARP group had a significantly higher short-term OM than the RP group (Table 2). We investigated the number of death events that occurred within 3 (as “operation-related”), and 12 months postoperatively. The HR between RP and RARP groups differed significantly by post-operative 3 (HR: 6.72, 2–22.63, *P* = 0.0021) and 12 months (HR: 5.5; 95% CI, 3.31–9.31, *P* < 0.0001). The 3- and 12-months post-op percentile differences in the death number of the RARP group in comparison with the total number of cases were about 0.63 and 2.92%, respectively. The 3- and 12-month post-op percentile differences in the death number in the RP group compared to that of the total number of patients were about 0.094 and 0.543%, respectively.

**Table 2:** Comparison of death patterns after RP, and RARP.

	By 3 months post-op					By 12 months post-op				
	RARP	RP	HR	95%CI	P_value	RARP	RP	HR	95%CI	P_value
Total cases (Total number in group: 3182)	20(0.628%)	3(0.094%)	6.72	2.00–22.63	0.0021	93(2.923%)	17(0.534%)	5.55	3.31–9.31	< .0001
2009 ~ 2012 (Total number in group: 1389)	14(1.007%)	1(0.07%)	13.99	1.84–106.33	0.0108	49(3.527%)	7(0.503%)	7.00	3.17–15.45	< .0001
2013 ~ 2017 (Total number in group: 1480)	11(0.743%)	1(0.067%)	11.22	1.45–86.91	0.0206	45(3.040%)	8(0.540%)	5.78	2.72–12.26	< .0001
Academic medical centers (Total number in group: 3090)	18(0.582%)	2(0.064%)	9.06	2.10–39.04	0.0031	92(2.977%)	15(0.485%)	6.18	3.58–10.67	< .0001
Big 4 (Total number in group: 562)	9(1.601%)	0(-)	-	-	-	38(6.761%)	4(0.711%)	10.01	3.57–28.04	< .0001

CI, confidence intervals; HR, hazard ratio; RARP, robot-assisted radical prostatectomy; RP, radical prostatectomy.

1 month post-op data are omitted because of the low number of death events for statistical analysis.

( ): proportion of death events represented as a percentile value of death events in each specific group.

Big 4 hospitals: Four academic medical institutes that had the largest volume of PCa surgeries during our entire observation period.

Notably, the number of deaths in the RARP group was reduced in the late observation period (year 2013–2017) compared to that in the early period (year 2009–2012) within 3- and 12-month post-op (Table 2). However, by 12 months post-op, the comparison of all-cause OM of RARP with that of RP ((HR: 5.779; 95% CI, 2.72–12.26, *P* < 0.0001) vs. (HR: 7.00; 95% CI, 3.17–15.45, *P* < 0.0001) for late vs. early) indicated a far worse prognosis for RARP in the early than in the late period. Within 3- and 12-month post-op, the percentile deaths in the RARP group at the ‘Big 4’ hospitals was worse than that of the total RARP patients (1.6% vs. 0.63%); (6.76% vs. 2.92%).

At the ‘Big 4’ hospitals, the risk of death as shown by the HR in the RARP group compared with those of RP group was the highest within 12 months post-operation (HR: 10.01; 95% CI, 3.57–28.04, *P* < 0.0001).

### Comparison of the secondary outcomes between groups

Regardless of when or where the surgeries were performed, compared with the RP group, the RARP group showed far fewer patients needing whole blood, packed red blood cells, and fresh frozen plasma transfusions within 1 month post-operation (Table S5). Wound disruptions occurred more frequently in the RP group than in the RARP group at 3- (HR: 0.152; 95% CI, 0.08–0.31, *P* < 0.0001) and 12-month post op (HR: 0.135; 95% CI, 0.07–0.26, *P* < 0.0001) (Table S6).

The HR of venous thromboembolism (VTE) between the RARP and RP groups was not statistically different within 3- and 12-months post op (Table S7). Renal failure occurred more frequently, in the RARP group than in the RP within 1 month post op (HR: 2.834; 95% CI, 1.42–5.64, *P* = 0.003) (Table S8). Pneumonia occurred more frequently, in the RARP group than in the RP in ‘Big 4’ hospitals within 1 month (HR: 12.129, 2.87–51.32, *P* = 0.0007) and 3 months post op (HR: 2.67, 1.37–5.2, *P* = 00.0039) (Table S9).

The comparison of shock events between the RP and RARP groups showed no significant difference within 3- (HR: 0.86; 95% CI, 0.6–1.25, *P* = 0.4373) or 12-month post op (HR: 0.92; 95% CI, 0.68–1.24, *P* = 0.5895) (Table S10). The RP and RARP groups also showed no significant differences in acute pyelonephritis events within 3- (HR: 0.998; 95% CI, 0.78–1.27, *P* = 0.9896) or 12-month post op (HR: 1.055; 95% CI, 0.84–1.32, *P* = 0.642) (Table S11).

Cardiopulmonary arrest (CPA) occurred more frequently in the RARP group than in the RP group within 12 months post op (HR: 7.572; 95% CI, 2.27–25.3, *P* = 0.001) (Table S12).

Notably, we did not observe that any evidence of modest differences in the secondary outcomes, investigated in our study, may have been directly responsible for the increased OM in the RARP group.

## Discussion

Most PCa patients in Korea opt for active surgical treatment^11^ and the introduction of robotic technology seems to have increased both the cost per surgical procedure and the volume of cases treated surgically^12^. Despite its wide popularity and concomitant high cost in Korea, our findings indicate that the RARP group had poorer short-term prognosis than the RP group in terms of all-cause OM.

When compared with the OM in the RP group, the OM of the RARP group from the ‘Big 4’ hospitals was higher than the national value within 3- and 12-months post-op. Indeed, the number of surgical treatments, especially RARP, in PCa patients is highly crowded in a limited number of medical institutes in Korea (Table S4).

Notably, we showed that the most deaths occurred after 12 months postoperatively. Considering the long natural history of PCa and relatively small death rates (about 2.92% of the total deaths) within 12 months post-op, that short-term mortality in the RARP group was higher than that in the RP group may not be universal, but limited events. A large number of surgeons and hospital volumes are known to be associated with better RARP outcomes^13^. However, we showed that the proportion of deaths in the RARP group was the highest in the ‘Big 4’ hospitals. This indicates a possibility that the greater centralization of RARP practices in the ‘Big 4’ hospitals may have been related to the higher OM in the RARP group.

Although RARP can be recommended for patients with locally-advanced PCa^14^, it has been performed even for very high-risk patients with cancer stage beyond T3b or in those with bone metastases^15^. The surgeon’s experience reportedly determines the operation time and blood loss in RARPs, whereas a positive surgical margin rate is determined mainly by the cancer stage, and not by the surgeon’s experience^16^. The study suggests a possibility that the prognosis of PCa patients after RARPs is more likely dependent on the pre-operational PCa stage, and not on the highly experienced surgeon’s skills.

The total amount spent on the care of PCa patients in Korea is increasing greatly. Hospitals that launched robotic surgery programs have an extensive and immediate increase in the use of robotic surgery, which is also associated with a decrease in traditional, far less expensive alternatives^1^, indicating fewer surgical choices for the patients. To compensate for the high installation and maintenance costs, hospitals have provided heavy incentives for the surgeons to perform RARPs instead of other alternative procedures in Korea^17^.

Efforts to compensate for the high costs of the robotic instrument may be responsible for its extensive access to the cancer lesions in advanced PCa patients^18^. That the short-term mortality rates in the ‘Big 4’ hospitals, with the largest surgical volumes, was higher than the national value, support this.

The relative number of deaths in RARP group were reduced in the early observation period (2009–2012) compared to that in the late observation period (2013–2017). This may have been due to our decade-long campaign for early prostate-specific antigen (PSA) screening and active surgical intervention in PCa patients in Korea.

Since the payment for RARP is not covered by the NHIS, a significant difference in the total cost between RARP and RP group have been shown in Korea^11^. While the direct medical cost for a single RARP (an exclusively “out-of-pocket” expense) ranges from 7,000 to 13,000 USD, the direct cost for RP (most of which is covered by the NHIS) ranges from USD 2,400 to 4,500, respectively, in Korea. Therefore, the actual cost difference between undergoing RARP and RP by patients in Korea is even higher than that in the United States, where the total costs for RARP and RP are approximately USD 14,000 and 10,100, respectively^19^.

Additionally, we discovered more frequent billing for blood transfusions in the RP group than in the RARP group. Wound disruptions were also more frequently observed in the RP group than in the RARP group, which is consistent with the findings reported in the study by Szu-Yuan Wu et al.^20^. The RARP group only showed modestly higher level of major surgical complications, including, renal failure, pneumonia, and CPA than RP group.

A limitation of this study is that we did not evaluate other major complications or confounding factors that may explain the unfavorable outcome in the RARP group. Our study was conducted based on health insurance claim data, which inherently limits the availability of clinically essential information, particularly those that can reflect the severity of the disease. This limitation introduces a potential selection bias, as we were unable to control for factors such as preoperative Gleason score or prostate-specific antigen levels. Additionally, due to the nature of the data, we could not identify specific causes of death at 3 months and 12 months postoperatively. Further studies with more comprehensive clinical data are needed to provide a more accurate understanding of the factors influencing mortality outcomes following prostatectomy. It is likely that robotic-assisted laparoscopic prostatectomy has increased for treatment of high-risk and high-stage prostate cancer.

In our study, we did not establish what exactly caused the poorer outcomes in the RARP group. This study also suggests a possibility that the prognosis of PCa patients after RARPs may have been dependent on unspecified condition of the patients or other surgical complications. Our study was conducted in Korea, a country with a high prevalence of robot-assisted surgery and relatively low costs, making it more accessible to the population. While our results may be more directly applicable to the Korean context, we believe they can provide valuable insights into the early changes that may occur in other countries as robotic surgeries become increasingly accessible and affordable. However, we acknowledge that caution must be exercised when generalizing our findings to other populations, as regional differences in healthcare systems and patient characteristics could influence the outcomes of prostatectomy procedures.

## Conclusions

The use of RARP appears not to have definitive short-term benefits other than less blood transfusion events and specific surgical complications in comparison with RP. Though the death number was relatively small, our data, indicating that RARP showed higher short-term mortality rate than RP, indicate a possibility that indications of RARP may have been extended to some advanced PCa patients. Considering its high cost and limited value, we recommend that the current indications for RARP in PCa patients be re-evaluated in Korea. This might be related with increase in robotic surgery in the elderly, and it is suggested that more meticulous measures will be needed for robotic surgery in the elderly.

## Supplementary Information

Supplementary Information.

## Data Availability

The datasets generated during and/or analyzed during the current study are available from the corresponding author on reasonable request.
