# Supplementary material for: Outcomes of prostate cancer patients after robot-assisted radical prostatectomy compared with open radical prostatectomy in Korea
Source: Sci Rep. 2023 May 15;13:7851. doi: 10.1038/s41598-023-34864-8 (PMC10185510; doi:10.1038/s41598-023-34864-8)
Supplement: Supplementary file 1 — Supplementary Information. [file 41598_2023_34864_MOESM1_ESM.pdf]

Supplementary materials

Outcomes of prostate cancer patients after robot-assisted radical prostatectomy compared with open radical prostatectomy in Korea

Jaehun Jung, M.D., Ph.D[1,2¶], Gi Hwan Bae, MS[2¶], Jae Heon Kim, M.D., Ph.D[3,4] and Jaehong Kim, M.D., Ph.D[5,*]

[1] Department of Preventive Medicine, Gachon University College of Medicine, Incheon, South Korea

[2] Artificial Intelligence and Big-Data Convergence Center, Gil Medical Center, Gachon University College of Medicine, Incheon, South Korea

[3] Department of Urology, Soonchunhyang University Hospital, Soonchunhyang University Medical College, Seoul, South Korea

[4] Urological Biomedicine Research Institute, Soonchunhyang University Seoul Hospital, Seoul, South Korea

[5] Department of Biochemistry, College of Medicine, Gachon University, Incheon, South Korea

¶ These authors contributed equally

Short title: Robot-assisted radical prostatectomy in Korea

***Corresponding Author**

Jaehong Kim

Department of Biochemistry, College of Medicine, Gachon University,

155 Gaetbeol-ro, Songdo-dong, Yeonsu-gu, Incheon 21999, Republic of Korea

Tel.: +82-32-899-6441

Fax: +82-32-899-6588

Email: geretics@gachon.ac.kr

Contents

Table S1. Diagnostic and procedure codes used to identify major complication after surgery.

Table S2. Codes used to identify chemotherapy, hormone-therapy, and radiation therapy

Table S3. International Classification of Diseases 10th Revision (ICD-10) mapping for Charlson Comorbidity Index

Table S4. Distribution of surgical events in hospital groups before PSM

Table S5. Comparison of transfusion events after RP, and RARP.

Table S6. Comparison of wound disruption patterns after RP, and RARP.

Table S7. Comparison of Venous thromboembolism patterns after RP, and RARP.

Table S8. Comparison of Renal failure patterns after RP, and RARP.

Table S9. Comparison of Pneumonia patterns after RP, and RARP.

Table S10. Comparison of Shock patterns after RP, and RARP.

Table S11. Comparison of acute pyelonephritis patterns after RP, and RARP.

Table S12. Comparison of cardiopulmonary arrest patterns after RP, and RARP.

Table S1. Diagnostic and procedure codes used to identify major complication after surgery.

Category	Diagnostic code	Procedure code
Intraoperative complications	Vascular injury (S350, S351, S352, S353, S354, S355, S357, S358, and S359)	Vessel repair (O2072, O2073, and O2074)
Postoperative complications	Lymphocele (I898)	-
	Lymphedema (I890)	-
	Bowel obstruction (K315, K565, K566, and K913)	-
Surgical complications	Wound disruption (T813)	Wound repair (SC027)
	Incisional hernia (K430, K431, K439, K450, K451, K458, K460, K461, and K469)	-
Blood transfusions	-	Whole blood (X6001, X6002, X1001, X1002, and B2050)
	-	Packed RBCs (X2021, X2022, X2031, X2032, X2091, X2092, X2111, X2112, X2131, X2132, X2512, and X2515)
	-	Fresh frozen plasma (X2011, X2012, X2041, X2042, X2051, X2052, X2071, X2072, X2141, X2142, X2504, X2505, and X2514)
	-	Platelet concentrate (X2081, X2082, X2121, X2122, X2511, and X2513)
Medical complications	Venous thromboembolism (I240, T801, I802, I822, I823, I828, I829, K550, I741, I742, I743, I744, I745, I748, I749, I260, and I269)	-
	Myocardial infarction (I210, I211, I212, I213, I2140, I2141, I2148, I2149, I219, I220, I221, I228, I229, and I252)	-
	Cardiopulmonary arrest (I460, I461, I469, R092, and R092)	-
	Respiratory failure (J960, J961, and J969)	-

	Renal failure (I129, I131, I132, N170, N172, N178, N179, N19, and N990)	-
	Bacteremia/sepsis (A400, A401, A402, A403, A408, A409, A410, A411, A412, A413, A414, A4150, A4151, A4152, A4158, A4159, A4180, A4188, A419, R651, T802, and T814) Shock (A419, A483, F430, R570, R571, R572, R578, R579, T780, T782, T794, T811, and T882)	-
	Shock (A419, A483, F430, R570, R571, R572, R578, R579, T780, T782, T794, T811, and T882)	-
	Pneumonia (A022, A548, B012, B250, B59, J100, J110, J120, J121, J122, J123, J128, J129, J13, J14, J150, J151, J152, J153, J154, J155, J156, J157, J158, J159, J160, J168, J170, J171, J172, J173, J178, J180,J181, J182, J188, J189, J678, J679, J680, J690, J691, J698, J700, J82, J8410, J8418, and J849)	-
	Delirium (F058 and F059)	-
	Acute pyelonephritis (N10)	-
	Stroke (G463, G464, I64, I60, I600, I601, I602, I603, I604, I605, I606, I606, I607, I608, I609, I6300, I6301, I6302, I6308, I6309, I6310, I6311, I6312, I6318, I6319, I6320, I6321, I6322, I6328, I6329, I6330, I6331, I6332, I6333, I6338, I6339, I6340, I6341, I6342, I6343, I6348, I6349, I6350, I6351, I6352, I6353, I6358, I6359, I636, I638, and I639)	-

Table S2. Codes used to identify chemotherapy, hormone-therapy, and radiation therapy

#	Mechanism	Agent	Code
**1**	Chemo-reagents: microtubule inhibitor	Docetaxel	148341BIJ (20 mg)<br>148342BIJ (80 mg)<br>148340BIJ (0.12g(20mg/mL))<br>148344BIJ (20mg(20mg/mL))<br>148348BIJ (80mg(20mg/mL))<br>148349BIJ 80mg(27.6mg/mL)<br>148346BIJ 20mg(40mg/mL)<br>148350BIJ 80mg(40mg/mL)<br>148351BIJ 0.12g(40mg/mL)
**2**		cabazitaxel	613901BIJ 60mg(40mg/mL)
**3**		paclitaxel	207830BIJ 30mg(6mg/mL)<br>207831BIJ 0.1g(6mg/mL)<br>207832BIJ 0.15g(6mg/mL)<br>207833BIJ 0.2g(6mg/mL)<br>207835BIJ 0.3g(6mg/mL)<br>503701BIJ 0.1g
**4**		vinBLAStine	247830BIJ 10mg(1mg/mL)
**5**		vinorelbine	248230BIJ 10mg(10mg/mL)<br>248231BIJ 50mg(10mg/mL)
**6**	Androgen receptor antagonist.	enzalutamide	627401ACS (40 mg)
**7**	Non-steroidal androgen receptor inhibitor. It competitively inhibits the action of androgens by binding to cytosol androgen receptors	bicalutamide	117201ATB (50 mg)<br>117202ATB 0.15g
**8**	For the treatment of advanced androgen-dependent carcinoma of the prostate (for palliation only)	estradiol valerate	155001ATB 1mg<br>155002ATB 2mg<br>297600ATB 2mg, 10 1t/pack<br>398400ATB 2mg, 16 1t/pack<br>433900ATB 1mg/2.5mg<br>434000ATB 1mg/5mg<br>434100ATB 2mg/3mg
**9**	A combination of estradiol with nitrogen mustard. stopping the cancerous cells from dividing into two new cells,	estramustine	155101ACH 0.14g
**10**	Noncrystalline mixtures of purified female sex hormones obtained either by its isolation from the urine of pregnant mares or by synthetic generation from vegetal material.	conjugated estrogens	155401ATB 0.3mg<br>155402ATB 0.625mg
**11**	Inhibiting 5α-reductase and thus preventing DHT production	finasteride	159001ATB 5mg

**12**	Immediate onset of action, binding to gonadotropin-releasing hormone (GnRH) receptors in the pituitary gland and blocking their interaction	degarelix	624401BIJ 88.2mg<br>624402BIJ 128mg
**13**	Gonadotropin Releasing Hormone Receptor Agonist.	goserelin acetate	167201BIJ 10.8mg<br>167202BIJ 3.6mg
**14**	GnRH agonist, acts as a potent inhibitor of gonadotropin secretion. suppression of ovarian and testicular steroidogenesis.	leuprolide acetate	182602BIJ 3.75mg<br>182604BIJ 11.25mg<br>182605BIJ 7.5mg<br>182606BIJ 22.5mg<br>182608BIJ 45mg<br>182610BIJ 30mg<br>182611BIJ 22.5mg<br>182630BIJ 14mg(5mg/mL)
**15**	Gonadotropin releasing hormone (GnRH) agonist that is a potent inhibitor of the synthesis of testosterone (in men)	triptorelin	244902BIJ 3.75mg<br>244930BIJ 0.105mg(0.105mg/mL)<br>467501BIJ 11.25mg<br>467502BIJ 22.5mg
**16**	Management of luteinizing hormone releasing hormone induced hot flashes in men with advanced prostate cancer	medroxyprogesterone	188903ATB 10mg<br>188906ATB 5mg<br>297600ATB 10mg<br>433900ATB 2.5mg<br>434000ATB 5mg<br>434100ATB 5mg<br>188905ATB 0.5g
**17**	Inhibitor of cytochrome P450 14α-demethylase (CYP17).	ketoconazole	179631CCM 0.2g(20mg/g)<br>179632CCM 0.3g(20mg/g)<br>179633CCM 0.4g(20mg/g)<br>179636CCM 10g(20mg/g)
**18**	Androgen biosynthesis inhibitor, that inhibits 17 a-hydroxylase/C17,20-lyase (CYP17)	abiraterone acetate	620402ATB (0.5g)
**19**	Synthetic progestin, inhibit intracellular androgen action. Secondary effects of megestrol acetate include direct cytotoxic effect at high concentration and inhibition of 5-alpha reductase, LH release, and adrenal axis.	megestrol acetate	189301ATB 40mg<br>189302ATB 0.16g<br>189303ASS 0.65g(0.125g/mL)<br>189330ASS 0.4g(40mg/mL)<br>189331ASS 0.8g(40mg/mL)<br>189335ASS 9.6g(40mg/mL)
**20**	Suppress adrenal androgens leading to a more complete androgen blockade	methylprednisolone	193302ATB 4mg<br>193305ATB 1mg<br>193601BIJ 0.125g<br>193603BIJ 40mg<br>193604BIJ 0.5g
**21**	Corticosteroids can suppress adrenal androgens leading to a more complete androgen blockade. 'Prior	prednisolone	193302ATB 4mg

	exposure before abiraterone’		193305ATB 1mg<br>217001ATB 5mg<br>217034ASY 0.5g(1mg/mL)<br>217035ASY 1.5g(3mg/mL)<br>193601BIJ 0.125g<br>193603BIJ 40mg<br>193604BIJ 0.5g<br>217302BIJ 0.25g
**22**	Suppresses the proliferation of T cells, B cells, and macrophages. It impairs antigen presentation and decreases the secretion of proinflammatory cytokines.	mitoxantrone	196530BIJ 23.3mg(2.33mg/mL)
**23**	Beneficial immunomodulatory effects in adaptive immunotherapy.	cyclophosphamide	139001ATB 50mg<br>139005BIJ 0.5g
**24**	Immunotherapy	BCG strain tice	114301BIJ (12.5 mg)
**25**	Inhibition of Topoisomerase I enzyme	topotecan	241901BIJ 4mg
**26**	Binds to HER2 and suppresses cancer cells growth, proliferation, and survival directly and indirectly	trastuzumab	242801BIJ 0.44g<br>242802BIJ 0.15g<br>242830BIJ 0.6g(0.12mg/mL)<br>626001BIJ 0.1g<br>626002BIJ 0.15g
**27**	Binding to DNA and interfering with its repair mechanism, eventually leading to cell death.	Cisplatin	134530BIJ10mg (0.5mg/mL)<br>134533BIJ 50mg(0.5mg/mL)<br>134534BIJ 50mg(1mg/mL)<br>184904ATB (200 mg)
**28**	Antiangiogenic effect on prostate cancer cell lines, mediated via the glucocorticoid receptor, leading to a reduction in vascular endothelial growth factor and interleukin-8 expression and has been shown to reduce expression of the androgen receptor (AR)	dexamethasone	141901ATB 0.5mg<br>141903ATB 0.75mg<br>142030BIJ 4mg(4mg/mL)<br>142230BIJ 4.37mg(4.37mg/mL)<br>142232BIJ 5mg(5mg/mL)<br>142233BIJ 20mg(5mg/mL)
**29**	Binds vascular endothelial growth factor (VEGF) to inhibit angiogenesis	bevacizumab	554330BIJ 0.1g(25mg/mL)<br>554331BIJ 0.4g(25mg/mL)
**30**	primarily related to intercalation of the planar ring with DNA and subsequent inhibition of DNA and RNA synthesis.	epirubicin	152730BIJ 10mg(2mg/mL)<br>152731BIJ 50mg(2mg/mL)
**31**	inhibits DNA synthesis and RNA synthesis, functions necessary for cells to survive.	melphalan	189901ATB 2mg<br>189902BIJ 50mg

Table S3. International Classification of Diseases 10th Revision (ICD-10) mapping for Charlson Comorbidity Index

Diseases	ICD-10 codes	Weight
Myocardial infarction	I21, I22, I252	1
Congestive heart failure	I43, I50, I099, I110, I130, I132, I255, I420, I425, I426, I427, I428, I429, P290	1
Peripheral vascular disease	I70, I71, I731, I738, I739, I771, I790, I792, K551, K558, K559, Z958, Z959	1
Cerebrovascular disease	G45, G46, I60 ~ I69, H340	1
Dementia	F00 ~ F03, G30, F051, G311	1
Chronic Obstructive Pulmonary Disease	J40 ~ J47, J60 ~ J67, I278, I279, J684, J701, J703	1
Connective Tissue Disease	M05, M32 ~ M34, M06, M315, M351, M353, M360	1
Peptic Ulcer Disease	K25 ~ K28	1
Mild Liver Disease	B18, K73, K74, K700, K701 ~ K703, K709, K717, K713, K714, K715, K760, K762 ~ K764, K768, K769, Z944	1
Diabetes without complications	E100, E101, E106, E108 ~ E111, E116, E118 ~ E121, E126, E128 ~ E131, E136, E138 ~ E141, E146, E148, E149	1
Diabetes with complications	E102 ~ E105, E107, E112 ~ E115, E117, E122 ~ E125, E127, E132 ~ E135, E137, E142 ~ E145, E147	2
Paraplegia and Hemiplegia	G81, G82, G041, G114, G801, G802, G830 ~ G834, G839	2
Renal Disease	N18, N19, N052 ~ N057, N250, I120, I131, N032 ~ N037, Z490, Z491, Z492, Z940, Z992	2
Cancer	C00 ~ C26, C30 ~ C34, C37 ~ C41, C43, C45 ~ C58, C60 ~ C76, C81 ~ C85, C88, C90 ~ C97	2
Moderate or Severe Liver Disease	K704, K711, K721, K729, K765, K766, K767, I850, I859, I864, I982	3
Metastatic Carcinoma	C77 ~ C80	6
Acquired immune deficiency syndrome	B20 ~ B22, B24	6

Table S4. Distribution of surgical events in hospital groups before PSM

	Total number of cases	RARP (12268)	RP (3233)	*P*
Group (I)	Top tier academic medical centers*	11587 (94.4%)	3232 (~100%)	<.0001
	Others	681	1	
Group (II)	Big 4 hospitals**	3478 (19.504%)	726 (28.350%)	<.0001
	Others	8790	2507	

RP, radical prostatectomy; RARP, robot-assisted radical prostatectomy.

* In Korea, the actual number of top tier academic medical centers varies slightly depending on how many get designated as "general hospital for the treatment of severely ill patients" every 3 years. Thereby, we presented their approximate number as 40.

** Big 4 hospitals: Four hospitals that had the largest volume of PCa surgeries during our entire observation period.

Table S5. Comparison of transfusion events after RP, and RARP.

	Whole blood					Packed RBCs					FFP				
	By 1 month post-op					By 1 month post-op					By 1 month post-op				
	RARP	RP	HR	95%CI	*P*	RARP	RP	HR	95%CI	*P*	RARP	RP	HR	95%CI	*P*
Tot Cases (Total number in group: 3182)	68 (2.14)	283 (8.9)	0.241	0.19-0.31	<.0001	67 (2.1)	282 (8.9)	0.239	0.18-0.31	<.0001	12 (0.38)	63(1.98)	0.191	0.1-0.35	<.0001
2009~2012 (Total number in group: 1389)	39 (2.8)	153 (11)	0.255	0.18-0.36	<.0001	38 (2.7)	153 (11)	0.248	0.17-0.35	<.0001	5(0.36)	29(2.09)	0.173	0.07-0.45	0.0003
2013~2017 (Total number in group: 1480)	29 (2)	96 (6.5)	0.305	0.2-0.46	<.0001	29 (2)	95 (6.4)	0.308	0.2-0.47	<.0001	6(0.41)	26(1.76)	0.233	0.1-0.57	0.0013
Big 4 (Total number in group: 562)	10 (1.8)	95 (17)	0.107	0.06-0.2	<.0001	11 (2)	96 (17)	0.116	0.06-0.22	<.0001	1(0.178)	25(4.49)	0.041	0.01-0.3	0.0017

CI, confidence intervals; HR, hazard ratio; RP, radical prostatectomy; RARP, robot-assisted radical prostatectomy; FFP, fresh frozen plasma; RBCs, Red blood cells;

* ( ): proportion of transfusion events represented as a percentile value of transfusion events in each specific group.

** Big 4 hospitals: Four hospitals that had the largest volume of PCa surgeries during our entire observation period.

Table S6. Comparison of wound disruption patterns after RP, and RARP.

	By 3 months post-op					By 12 months post-op				
	RARP	RP	HR	95%CI	*P*	RARP	RP	HR	95%CI	*P*
Tot Cases (Total number in group: 3182)	9(0.28)	60(1.86)	0.152	0.08-0.31	<.0001	10 (0.31)	77(2.42)	0.135	0.07-0.26	<.0001
2009~2012 (Total number in group: 1389)	5(0.26)	25(1.80)	0.202	0.08-0.53	0.0011	6(0.43)	31(2.23)	0.2	0.08-0.48	0.0003
2013~2017 (Total number in group: 1480)	4(0.27)	27(1.82)	0.152	0.05-0.43	0.0004	6(0.41)	34(2.30)	0.186	0.08-0.44	0.0001
Big 4 (Total number in group: 562)	3(0.53)	12(2.14)	0.26	0.07-0.92	0.0371	3(0.53)	14(2.49)	0.241	0.07-0.84	0.0252

CI, confidence intervals; HR, hazard ratio; RP, radical prostatectomy; RARP, robot-assisted radical prostatectomy.

* ( ): proportion of wound disruption events represented as a percentile value of the events in each specific group.

** Big 4 hospitals: Four hospitals that had the largest volume of PCa surgeries during our entire observation period.

Table S7. Comparison of Venous thromboembolism patterns after RP, and RARP.

	By 3 months post-op					By 12 months post-op				
	**RARP**	**RP**	**HR**	**95%CI**	***P***	**RARP**	**RP**	**HR**	**95%CI**	***P***
Tot Cases (Total number in group: 3182)	20(0.63)	20(0.63)	1.013	0.55-1.88	0.9663	44(1.38)	45(1.41)	1.015	0.67-1.54	0.9446
2009~2012 (Total number in group: 1389)	7(0.50)	6(0.43)	0.668	0.11-4	0.6586	16(1.15)	19(1.37)	1.175	0.39-3.5	0.7722
2013~2017 (Total number in group: 1480)	12(0.81)	14(0.95)	0.88	0.41-1.9	0.7442	21(1.42)	27(1.82)	0.818	0.46-1.45	0.4905
Big 4 (Total number in group: 562)	7(1.25)	3(0.53)	2.428	0.63-9.39	0.1985	14(2.49)	8(1.42)	1.961	0.82-4.68	0.1285

CI, confidence intervals; HR, hazard ratio; RP, radical prostatectomy; RARP, robot-assisted radical prostatectomy.

* ( ): proportion of venous thromboembolism events represented as a percentile value of the events in each specific group.

** Big 4 hospitals: Four hospitals that had the largest volume of PCa surgeries during our entire observation period.

Table S8. Comparison of Renal failure patterns after RP, and RARP.

	By 1 month post-op					By 3 months post-op				
	**RARP**	**RP**	**HR**	**95%CI**	***P***	**RARP**	**RP**	**HR**	**95%CI**	***P***
Tot Cases (Total number in group: 3182)	32(1.01)	11(0.35)	2.834	1.42-5.64	0.003	40(1.26)	23(0.72)	1.762	1.05-2.94	0.0304
2009~2012 (Total number in group: 1389)	13(0.94)	3(0.22)	4.342	1.24-15.24	0.0219	18(1.30)	9(0.65)	2.014	0.9-4.48	0.0863
2013~2017 (Total number in group: 1480)	11(0.74)	5(0.34)	2.219	0.77-6.39	0.1395	16(1.08)	10(0.68)	1.639	0.74-3.61	0.2203
Big 4 (Total number in group: 562)	12(2.14)	4(0.71)	3.032	0.98-9.4	0.0547	14(2.49)	8(1.42)	1.815	0.76-4.33	0.1786

CI, confidence intervals; HR, hazard ratio; RP, radical prostatectomy; RARP, robot-assisted radical prostatectomy.

* ( ): proportion of renal failure events represented as a percentile value of events in each specific group.

** Big 4 hospitals: Four hospitals that had the largest volume of PCa surgeries during our entire observation period.

Table S9. Comparison of Pneumonia patterns after RP, and RARP.

	By 1 month post-op					By 3 months post-op				
	**RARP**	**RP**	**HR**	**95%CI**	***P***	**RARP**	**RP**	**HR**	**95%CI**	***P***
Tot Cases (Total number in group: 3182)	38(1.29)	21(0.66)	1.818	1.07-3.1	0.0279	73(2.29)	50(1.57)	1.478	1.03-2.12	0.0334
2009~2012 (Total number in group: 1389)	20(1.44)	7(0.50)	2.859	1.21-6.76	0.0167	36(2.59)	22(1.58)	1.643	0.97-2.79	0.0664
2013~2017 (Total number in group: 1480)	10(0.68)	10(0.68)	1.008	0.42-2.42	0.9854	22(1.49)	23(1.55)	0.979	0.55-1.76	0.942
Big 4 (Total number in group: 562)	24(4.27)	2(0.36)	12.129	2.87-51.32	0.0007	31(5.51)	12(2.14)	2.67	1.37-5.2	0.0039

CI, confidence intervals; HR, hazard ratio; RP, radical prostatectomy; RARP, robot-assisted radical prostatectomy.

* ( ): proportion of pneumonia events represented as a percentile value of events in each specific group.

** Big 4 hospitals: Four hospitals that had the largest volume of PCa surgeries during our entire observation period.

Table S10. Comparison of Shock patterns after RP, and RARP.

	By 3 months post-op					By 12 months post-op				
	**RARP**	**RP**	**HR**	**95%CI**	***P***	**RARP**	**RP**	**HR**	**95%CI**	***P***
Tot Cases (Total number in group: 3182)	52(1.63)	61(1.92)	0.864	0.6-1.25	0.4373	82(2.58)	92(2.89)	0.921	0.68-1.24	0.5895
2009~2012 (Total number in group: 1389)	20(1.44)	26(1.87)	0.774	0.43-1.39	0.3883	32(2.30)	42(3.02)	0.781	0.49-1.24	0.2925
2013~2017 (Total number in group: 1480)	10(0.68)	28(1.89)	0.367	0.18-0.76	0.0065	25(1.69)	38(2.57)	0.694	0.42-1.15	0.1556
Big 4 (Total number in group: 562)	7(1.25)	18(3.20)	0.406	0.17-0.97	0.0428	12(2.14)	24(4.27)	0.563	0.28-1.13	0.1039

CI, confidence intervals; HR, hazard ratio; RP, radical prostatectomy; RARP, robot-assisted radical prostatectomy.

* ( ): proportion of shock events represented as a percentile value of the events in each specific groups.

** Big 4 hospitals: Four hospitals that had the largest volume of PCa surgeries during our entire observation period.

Table S11. Comparison of acute pyelonephritis patterns after RP, and RARP.

	By 3 months post-op					By 12 months post-op				
	**RARP**	**RP**	**HR**	**95%CI**	***P***	**RARP**	**RP**	**HR**	**95%CI**	***P***
Tot Cases (Total number in group: 3182)	130(4.09)	132(4.15)	0.998	0.78-1.27	0.9896	153(4.81)	151(4.75)	1.055	0.84-1.32	0.642
2009~2012 (Total number in group: 1389)	46(3.31)	48(3.46)	0.967	0.65-1.45	0.8696	55(3.96)	53(3.82)	1.069	0.73-1.56	0.7298
2013~2017 (Total number in group: 1480)	82(5.54)	74(5.00)	1.135	0.83-1.55	0.4295	94(6.35)	86(5.81)	1.151	0.86-1.54	0.3451
Big 4 (Total number in group: 562)	24(4.27)	27(4.80)	0.927	0.53-1.61	0.7872	33(5.87)	33(5.87)	1.117	0.69-1.81	0.6528

CI, confidence intervals; HR, hazard ratio; RP, radical prostatectomy; RARP, robot-assisted radical prostatectomy.

* ( ): proportion of acute pyelonephritis events represented as a percentile value of the events in each specific groups.

** Big 4 hospitals: Four hospitals that had the largest volume of PCa surgeries during our entire observation period.

Table S12. Comparison of cardiopulmonary arrest patterns after RP, and RARP.

	By 3 months post-op					By 12 months post-op				
	**RARP**	**RP**	**HR**	**95%CI**	***P***	**RARP**	**RP**	**HR**	**95%CI**	***P***
Tot Cases (Total number in group: 3182)	8(0.25)	1(0.03)	8.092	1.01-64.7	0.0487	22(0.69)	3(0.09)	7.572	2.27-25.3	0.001
2009~2012 (Total number in group: 1389)	6(0.50)	0(-)				10(0.72)	2(0.14)	5.125	1.12-23.39	0.0349
2013~2017 (Total number in group: 1480)	3(0,20)	1(0.07)	3.071	0.32-29.52	0.3312	9(0.61)	1(0.07)	9.403	1.19-74.22	0.0335
Big 4 (Total number in group: 562)	2(0.36)	0(-)				6(1.07)	1(0.18)	6.665	0.8-55.34	0.079

CI, confidence intervals; HR, hazard ratio; RP, radical prostatectomy; RARP, robot-assisted radical prostatectomy.

* ( ): proportion of cardiopulmonary arrest events represented as a percentile value of the events in each specific groups.

** Big 4 hospitals: Four hospitals that had the largest volume of PCa surgeries during our entire observation period.
